# Uncovering the transcriptomic and epigenomic landscape of nicotinic receptor genes in non-neuronal tissues

**DOI:** 10.1186/s12864-017-3813-4

**Published:** 2017-06-05

**Authors:** Bo Zhang, Pamela Madden, Junchen Gu, Xiaoyun Xing, Savita Sankar, Jennifer Flynn, Kristen Kroll, Ting Wang

**Affiliations:** 1 Center of Regenerative Medicine, Department of Developmental Biology, Washington University School of Medicine, Room 3212, 4515 McKinley Research Building, 4515 McKinley Ave, St. Louis, MO 63110 USA; 2 Center for Genome Sciences and Systems Biology, Department of Genetics, Washington University School of Medicine, Room 5211, 4515 McKinley Research Building, 4515 McKinley Ave, St. Louis, MO 63110 USA; 3 Department of Psychiatry, Washington University School of Medicine, St. Louis, MO 63110 USA

**Keywords:** Nicotinic acetylcholine receptors, CHRNA4, Epigenetics, Tissue-specificity, Liver, Evolution

## Abstract

**Background:**

Nicotinic acetylcholine receptors (nAChRs) play an important role in cellular physiology and human nicotine dependence, and are closely associated with many human diseases including cancer. For example, previous studies suggest that nAChRs can re-wire gene regulatory networks in lung cancer cell lines. However, the tissue specificity of nAChRs genes and their regulation remain unexplored.

**Result:**

In this study, we integrated data from multiple large genomic consortiums, including ENCODE, Roadmap Epigenomics, GTEx, and FANTOM, to define the transcriptomic and epigenomic landscape of all nicotinic receptor genes across many different human tissues and cell types. We found that many important nAChRs, including *CHRNA3*, *CHRNA4*, *CHRNA5,* and *CHRNB4*, exhibited strong non-neuronal tissue-specific expression patterns. *CHRNA3*, *CHRNA5*, and *CHRNB4* were highly expressed in human colon and small intestine, and *CHRNA4* was highly expressed in human liver. By comparing the epigenetic marks of *CHRNA4* in human liver and hippocampus, we identified a novel liver-specific transcription start site (TSS) of *CHRNA4.* We further demonstrated that *CHRNA4* was specifically transcribed in hepatocytes but not transcribed in hepatic sinusoids and stellate cells, and that transcription factors *HNF4A* and *RXRA* were likely upstream regulators of *CHRNA4*. Our findings suggest that *CHRNA4* has distinct transcriptional regulatory mechanisms in human liver and brain, and that this tissue-specific expression pattern is evolutionarily conserved in mouse. Finally, we found that liver-specific *CHRNA4* transcription was highly correlated with genes involved in the nicotine metabolism, including *CYP2A6*, *UGT2B7*, and *FMO3*. These genes were significantly down-regulated in liver cancer patients, whereas *CHRNA4* is also significantly down-regulated in cancer-matched normal livers.

**Conclusions:**

Our results suggest important non-neuronally expressed nicotinic acetylcholine receptors in the human body. These non-neuronal expression patterns are highly tissue-specific, and are epigenetically conserved during evolution in the context of non-conserved DNA sequence.

**Electronic supplementary material:**

The online version of this article (doi:10.1186/s12864-017-3813-4) contains supplementary material, which is available to authorized users.

## Background

Tobacco dependence (mainly through cigarette smoking) is a major global health problem and is a main cause of cancer and cancer-related death throughout the world. Nicotine, the biologically active substance in tobacco, promotes the addiction of smoking behaviors through activation of nicotinic acetylcholine receptors (nAChRs) [1]. These nAChRs typically combine to form fast, ionotropic cationic nicotinic receptor channels. Pentameric nAChRs usually consist of five subunits, with an overall molecular weight of 290 kDa. nAChRs subunits are broadly classified into two subtypes: muscle-type nicotinic receptors, including α1, β1, γ, δ, and ε subunits, and neuronal-type nicotinic receptors, including α2 − α10 and β2 − β4 subunits. Neuronal-type nicotinic receptors are usually found in the brain, and exhibit some similarities with GABAa receptors and glycine receptors [1]. In human brain, the α4 and β2 subunits are predominantly expressed and form pentameric (α4)_3_(β2)_2_ and (α4)_2_(β2)_3_ nAChRs. Other nAChR subunits, including α3, α5 α7, β3, and β4 are also expressed in human brain, usually forming homomeric and heteromeric receptors [2].

Neuronal-type nAChRs are generally believed to function in the brain and contribute to nicotine dependence through reward pathways [3]. Interestingly, previous studies reported that several neuronal-type nAChRs are also expressed in lung cancer cells and intestinal epithelium cells [4–7]. However, the overall expression pattern of nAChRs in different human tissues is largely unknown. To gain the knowledge of tissue - specific regulation of nAChRs, we took advantage of resources generated by several large genomic consortiums that aim to functionally annotate the human genome, including the ENCODE project [8], Roadmap Human Epigenomics project [9], FANTOM project [10], and GTEx project [11]. By comparing and combining extensive genomic datasets produced by these consortiums, we were able to define a comprehensive transcriptomic and epigenomic landscape of nAChR genes and investigate the regulatory mechanisms governing activities of these important genes.

Surprisingly, our investigation revealed that many neuronal-type nicotinic receptor subunits were highly expressed in non-neuronal tissues. In particular, we identified liver-specific expression of *CHRNA4*, and colon- and intestine-specific expression of *CHRNA3*, *CHRNA5*, and *CHRNB4*. These tissue-specific expression patterns of nAChRs were consistent with tissue-specific epigenetic patterns of these genes. Additionally, we discovered a novel alternative promoter of *CHRNA4* in human liver, through which transcription factors *HNF4A* and *RXRA* could directly regulate *CHRNA4* expression in hepatocytes. Despite the lack of DNA sequence conservation at the liver-specific promoter of CHRNA4 between rodents and hominoids, the liver-specific expression and regulatory mechanism of CHRNA4 seem to be evolutionarily conserved between human and mouse liver. These results suggest a genetically dynamic but epigenetically conserved evolutionary history of *CHRNA4*.

## Results

### Tissue-specific expression pattern of human nAChRs

**Fig. 1:**
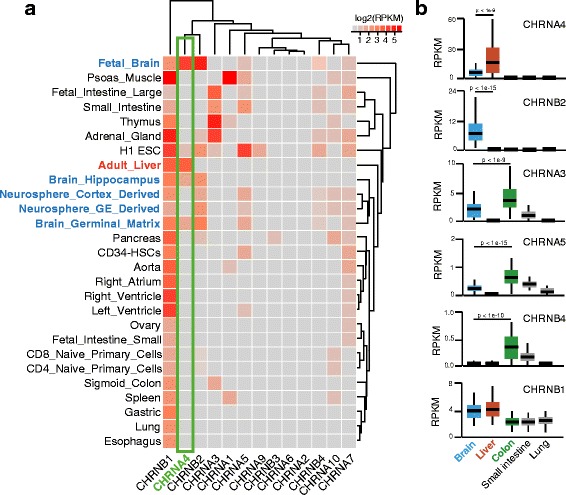
Tissue-specific expression patterns of nicotinic acetylcholine receptors (nAChRs). **a** Heat map view of nAChRs expression patterns in 27 human tissue/cell types. *Color scale* indicates the expression level (RPKM) measured by RNA-seq from the Human Roadmap Epigenome project. **b** Expression level of *CHRNA4*, *CHRNB2*, *CHRNA3*, *CHRNA5*, *CHRNAB4*, and *CHRNB1* in human brain, liver, colon, small intestine, and lung tissue. Y-axis indicates expression level (RPKM) measured by RNA-seq data from the GTEx project. Student *t*-test was performed to detect statistical significance

To understand the expression pattern of nAChR subunits in human, we examined the mRNA expression levels of 13 nAChRs genes that encode 9 *alpha*-subunits and 4 *beta*-subunits. By analyzing mRNA-sequencing data from 27 different human tissues and cell types generated by the Roadmap Epigenomes project [9], we found that nAChRs varied widely in their expression in a tissue-dependent manner. For example, *CHRNB1* was found to be highly expressed across multiple tissues (Fig. 1a). Surprisingly, while *CHRNB2*, the beta-subunit of α4β2-containing nicotinic receptors, was found to only be highly expressed in brain tissues, *CHRNA4*, the most abundant nAChR alpha-subunit in human brain [2], was highly expressed in human adult liver in addition to being highly expressed in brain (Fig. 1). This tissue-specific expression pattern was validated in an independent cohort based on the Genotype-Tissue Expression project (GTEx) [11] (Fig. 1b).

The *CHRNA3*-*CHRNA5*-*CHRNB4* loci (chr15-q25.1) is the hotspot for genetic variants that are associated with heavy smoking and nicotine dependence [12–14]. While these genes exhibit expected expression in the brain, we found much higher expression levels of *CHRNA3*, *CHRNA5*, and *CHRNB4* in colon and small intestine than in brain tissues. This expression pattern was also recapitulated by the GTEx datasets (Fig. 1b).

### Epigenetic profile predicts novel liver-specific alternative promoter for *CHRNA4*

**Fig. 2:**
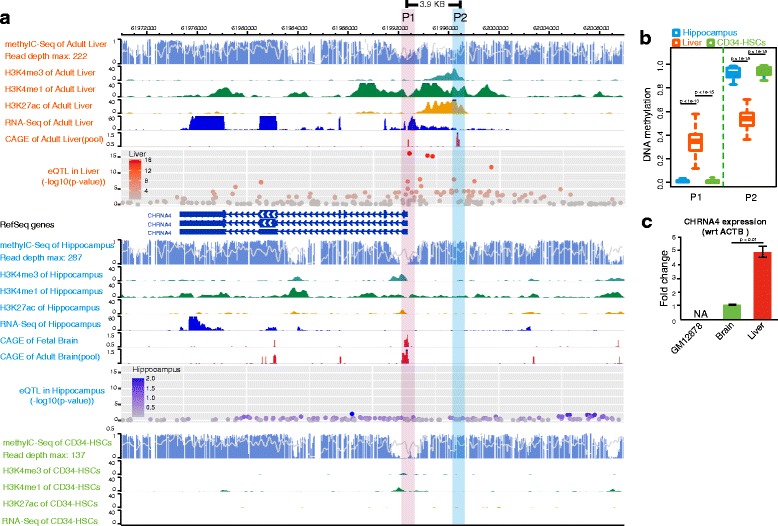
Epigenetic landscape of *CHRNA4* in human. **a** Epigenetic landscape and transcription signal around *CHRNA4* in human liver, hippocampus, and CD34 hematopoietic stem cells. The RefSeq promoter of *CHRNA4* (P1, *pink-shaded*) shows enrichment of H3K4me3 and H3K27ac histone modifications, especially in human hippocampus. CAGE-seq signal of fetal brain indicates that *CHRNA4* was transcribed from RefSeq canonical promoter P1. Liver-specific promoter P2 (*blue-shade*), which is located ~3.9 KB upstream of the RefSeq promoter, is enriched for strong, active histone modifications. The CAGE-seq signal of adult liver indicates *CHRNA4* is transcribed from the liver-specific promoter P2. **b** Averaged DNA methylation levels of promoters P1 and P2 in human liver, hippocampus, and CD34 hematopoietic stem cells. Mann-Whitney U test was performed to detect statistical significance. **c** Relative expression level of *CHRNA4* measured by qRT-PCR in human GM12878 cell line, brain, and liver. Student *t*-test was performed to detect statistical significance

Tissue-specific epigenetic profiles of a gene are strong predictors of tissue-specific gene activity. Active histone modifications (for example, H3K4me1, H3K4me3, and H3K27ac) and DNA hypomethylation in promoter regions are hallmarks of active genes [8, 9, 15]. To understand the high expression of neuronal-type nAChRs in human non-neuronal tissues, we examined the epigenetic landscape around *CHRNA3*, *CHRNA4*, *CHRNA5*, *CHRNB2*, and *CHRNB4* in human liver, hippocampus, CD34 hematopoietic stem cells, colon, and lung tissues using the WashU Epigenome browser [16, 17]. We found that tissue-specific expression of nAChRs was strongly associated with the tissue-specific active epigenetic marks around the gene promoter (Additional file 1: Figure S1). In human hippocampus, we detected strong H3K4me3 and H3K27ac signals around known transcription start sites (TSS) of *CHRNA3*, *CHRNA5*, *CHRNB2*, *CHRNB4*, and *CHRNA4* (Additional file 2: Figure. S2). Strikingly, in liver we detected very strong H3K4me3 and H3K27ac signals at 3.9 kb upstream of the known RefSeq TSS (Fig. 2a). This stunning promoter signature predicted a liver-specific, alternative promoter and/or transcription start site for CHRNA4.

Our prediction was confirmed using the Cap Analysis Gene Expression sequencing (CAGE-seq) data from the FANTOM5 project [10]. CAGE-seq identifies gene transcription start sites by sequencing the 5′ capped ends of mRNAs [18]. We found that the CAGE signal from human liver was only presented at −3.9 kb upstream (chr20: 61,996,626–61,996,696) of the canonical TSS. In contrast, CAGE signal from brain was located around the known canonical *CHRNA4* RefSeq TSS (chr20: 61,992,747–61,992,748) (Fig. 2a). As a control, we examined the histone modifications and CAGE signal for the *CHRNA4* gene in human CD34^+^ hematopoietic stem cells (CD34-HSCs), where the gene is known to be silent (Fig. 1a). We did not observe enrichment of active histone modification marker (H3K27ac) at either brain-specific or liver-specific promoter regions of *CHRNA4* in HSCs, nor did we observe CAGE signal in these regions (Fig. 2a). We also noticed that the single nucleotide polymorphism (SNPs) around *CHRNA4* were not associated with CHRNA4 expression level in brain hippocampus, but 4 SNPs were strongly associated with *CHRNA4* expression level in human liver (Fig. 2a, processed expression quantitative trait loci (eQTL) data was downloaded from GTEx Project).

We also examined the DNA methylation level of both brain-specific and liver-specific *CHRNA4* promoter regions (+/−500 bp of the TSS). The liver-specific *CHRNA4* promoter was significantly hypomethylated in human liver and hypermethylated in both brain and CD34-HSCs (Fig. 2b), while the brain-specific promoter was hypermethylated in liver and hypomethylated in the hippocampus and CD34-HSCs (Fig. 2b). Additionally, we validated the expression level of CHRNA4 using q RT-PCR, and confirmed that the expression of CHRNA4 is about five-fold higher in human liver than it is in human brain, and *CHRNA4* is not expressed in B cell lymphocyte (Fig. 2c). Taken together, our results reveal a distinctive promoter usage of the *CHRNA4* gene in human liver and brain, highlighting a novel tissue-specific regulatory mechanism.

### Conserved expression and epigenetic patterns of *Chrna4* in mouse

Nicotinic acetylcholine receptors play important roles in the central nervous system, and are highly conserved from Drosophila to vertebrates [19]. We next determined if the unexpected liver-specific expression of *CHRNA4* observed in human was an evolutionarily conserved phenomenon. To this end we took advantage of the data resources produced by the mouseENCODE consortium and FANTOM5 [10, 20] by integrating gene expression data, epigenomic data, and RNA polymerase II (Pol-2) ChIP-seq data, with CAGE-seq data from mouse brain and liver. We found that the epigenetic landscape between human and mouse is highly conserved in a tissue-specific manner surrounding the *CHRNA4* /*chrna4* gene in human and mouse, respectively. In mouse liver, active epigenetic modifications, including the H3K27ac signal, PoI-2 ChIP-seq signals, and DNaseI hypersensitivity signal, were highly enriched in a region ~4.8 kb upstream of the RefSeq annotated *Chrna4* TSS. In contrast, in mouse brain, the active epigenetic modifications were depleted in this region, but enriched around the canonical promoter (Additional file 2: Figure S2A). CAGE-seq data also support the alterative TSS usages between brain and liver (Additional file 2: Figure S2A, Fig. 4b). Specifically, the CAGE-seq signals were not found in hepatic sinusoids and stellate cells but was only presented in hepatocytes (Fig. 4b). We further confirmed the higher expression of *Chrna4* in mouse liver than in brain with RT-PCR (Additional file 2: Fig. S2B). These data strongly suggest that there exists a novel but evolutional conserved mechanism to regulate tissue-specific activities of *CHRNA4*/*Chrna4* in human and mouse, and that this neuronal-type nAChR might play a conserved and uncharacterized role in liver.

**Fig. 3:**
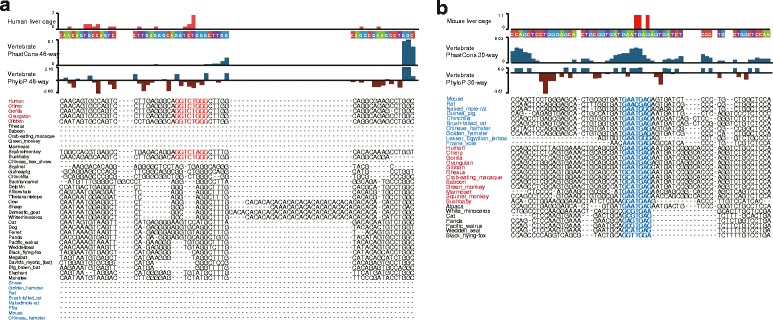
Evolutionary dynamics of the *CHRNA4* liver-specific promoter. **a** WashU EpiGenome Browser views of the conserved *CHRNA4* liver-specific promoter in human and the orthologous sequence alignment in other vertebrate animals. The species without an orthologous sequence in this region were not displayed (except primates and rodents). **b** WashU EpiGenome Browser views of the conserved *CHRNA4* liver-specific promoter in mouse and the orthologous sequence alignment in other vertebrate animals. The species without an orthologous sequence in this region were not displayed

**Fig. 4:**
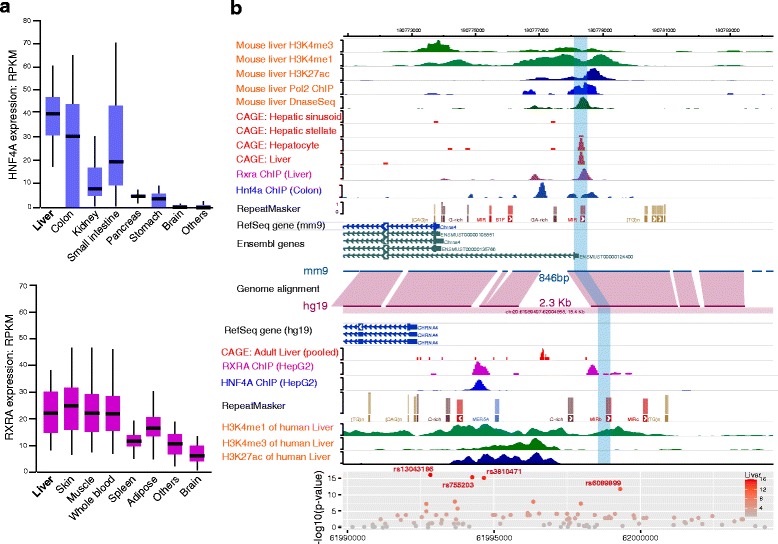
Regulation of *CHRNA4* by *HNF4* and *RXRA*. **a** *HNF4A* (*top*) and *RXRA* (*bottom*) are highly expressed in human liver as compared to other human tissues. The *Y-axis* indicates expression level (RPKM) measured by RNA-seq data from the GTEx project. The tissues are ranked by median expression of *HNF4A* and *RXRA*. **b** Evolutionarily conserved epigenetic landscape and transcriptional pattern of Chrna4 in mouse and human liver. The liver-specific promoter (*blue-shade*), which is located ~4.8 KB upstream of the RefSeq promoter, is enriched for strong, active histone modifications and RNA polymerase II ChIP-seq signal (Pol2). The CAGE-seq signal of liver indicates Chrna4 is predominantly transcribed in hepatocyte from the liver-specific promoter P2. Available ChIP-seq data indicate that *HNF4A/Hnf4a* and *RXRA/Rxra* directly bind to the liver-specific promoter of *CHRNA4 /Chrna4* in human and mouse liver, respectively

Further, we checked the DNA sequence conservation of the liver-specific *CHRNA4* and Chrna4 promoters. We found that the orthologous region of the human liver-specific *CHRNA4* TSS is conserved only in hominoid monkeys, and is absent in many other monkeys (Rhesus, Baboon, macaque, and marmoset) and rodents (Fig. 3a). Conversely, the orthologous region of the mouse liver-specific *Chrna4* TSS is highly conserved among rodents, primates and other mammals (Fig. 3b). In the human genome, we found a highly enriched H3K4me1 signal in the orthologous region of the mouse liver-specific *Chrna4* TSS, which is located ~2 kb upstream the human liver-specific *CHRNA4* TSS (Fig. 2a, Fig. 4b). Such evidence suggests that a ‘turn-over’ event may have occurred during primate evolution, and may also suggest that the ‘evolutionarily conserved’ liver-specific expression of *CHRNA4*/*Chrna4* evolved independently in hominoids and rodent animals.

### HNF4A and RXRA may be involved in liver-specific *CHRNA4* expression

To understand the liver-specific regulatory mechanism of *CHRNA4*, we examined the transcription factor binding events around the *CHRNA4* promoter region. Over 20 different transcription factors were found to bind to the 10 kb region surrounding the *CHRNA4* promoter, as determined by the ENCODE consortium (Additional file 3: Figure S3). Considering the liver-specific expression pattern of *CHRNA4*, we reasoned that the upstream transcription factors of *CHRNA4* should have a similar liver-specific expression pattern. After examining the expression patterns of 22 transcription factors that had binding sites near the *CHRNA4* promoter across 31 major human tissues, we identified *HNF4A* and *RXRA* as highly expressed in human livers (Fig. 4a) and with binding sites in the vicinity of the *CHRNA4* promoter. We also analyzed ChIP-seq data for *Hnf4a* and *Rxra* in mouse, and found that *Hnf4a* and *Rxra* directly bind to the promoter region of *Chrna4*. Furthermore, an *Rxra* ChIP-seq peak directly overlapped with the mouse liver-specific TSS (Fig. 4b). These data indicate that *HNF4A /Hnf4a* and *RXRA/Rxra* could be important TFs regulating the liver-specific expression of *CHRNA4 / Chrna4* in both human and mouse, providing a potential mechanistic explanation behind the observed ‘conserved expression pattern’ of *CHRNA4/Chrna4* between rodents and primates. We identified 4 SNPs to be significantly associated with expression of *CHRNA4* in human liver (eQTL), and all 4 SNPs were located within a liver - specific regulatory element (Figs. 2a and 4b). Two of the SNPs, rs755203 and rs3810471, were directly under *RXRA* and *HNF4A* ChIP-seq peaks, although they did not overlap with predicted RXRA or HNF4A binding motifs. Three SNPs, rs6089899, rs755203, and rs3810471, were predicted to influence binding affinities of several transcription factors including Krüppel-Like Factor (KLF) family members (Table 1).

**Table 1:**
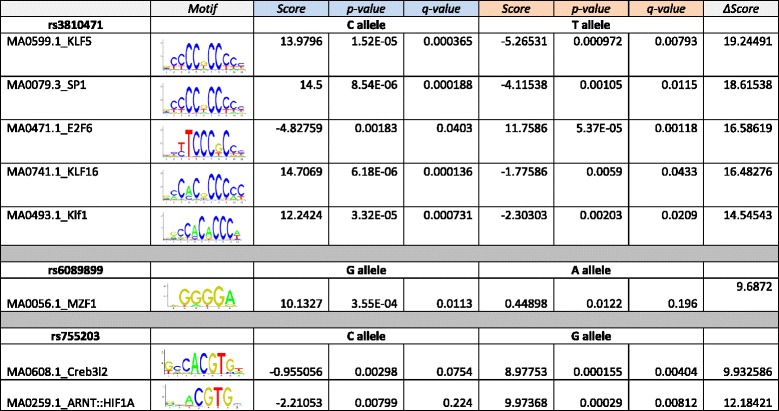
Transcription factor motif analysis of SNPs associated with *CHRNA4* liver-specific expression.

### Liver-specific *CHRNA4* expression is associated with nicotine metabolism pathway

**Fig. 5:**
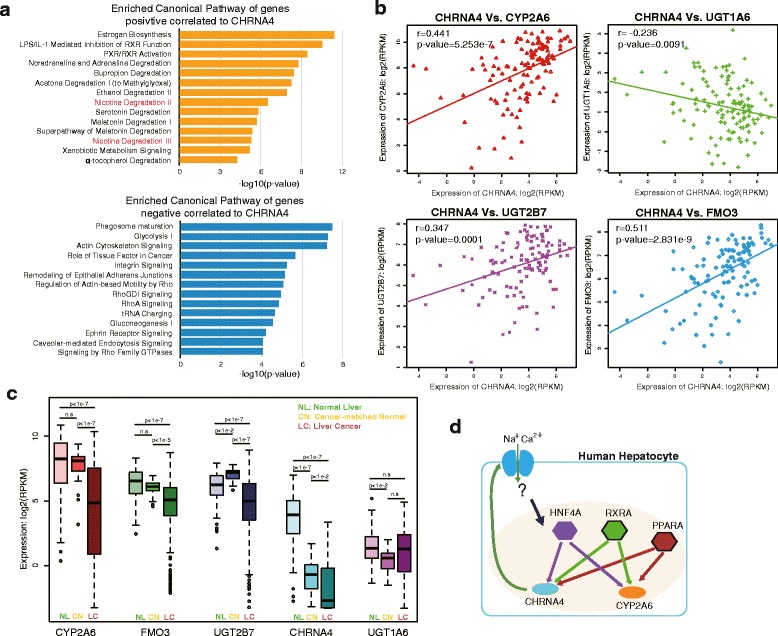
Correlated expression between *CHRNA4* and *CYP2A6*. **a** The enriched Canonical Pathways of genes positively/negatively correlated with CHRNA4 in 119 normal liver samples. **b** Expression Pearson correlation coefficient between *CHRNA4* and nicotine-metabolism related genes. **c** *CHRNA4* and nicotine-metabolism related genes were significantly down-regulated in hepatocellular carcinoma compared to normal liver samples. ANOVA was performed to detect statistical significance. **d** Module of correlation and regulation between *CHRNA4* and *CYP2A6* in human hepatocytes. *CHRNA4* and *CYP2A6* are co-regulated by *HNF4A* and *RXRA*. The *CHRNA4* nicotinic receptor on the cell membrane may trigger downstream signaling pathways to stimulate regulation of *CYP2A6* and itself

To understand the potential roles of *CHRNA4* in the liver, we investigated the enriched functions of genes co-expressed with *CHRNA4* in 119 normal human liver samples (GTEx V6). We found 705 genes were significantly and positively correlated with *CHRNA4* expression, whereas another 380 genes were significantly and negatively correlated (Additional file 4: Figure S4, Additional file 5: Table S1). By using Ingenuity Pathway Analysis, we found that genes significantly positively correlated with *CHRNA4* were highly enriched in several metabolic pathways, specifically in nicotine degradation (Fig. 5a, Additional file 6: Fig. S5). We further examined the expression level of important nicotine metabolism genes, and found expression of *CYP2A6*, *UGT2B7*, and *FMO3* were significantly correlated with *CHRNA4*’s expression in human liver (Fig. 5b). *UGT1A6* exhibited anti-correlation but the correlation was less significant (Fig. 5b) and the expression level was relatively low (Additional file 7: Figure S6).

Smoking is generally believed to be a risk factor for liver cancer [21], we further examined the expression of *CHRNA4* and nicotine metabolism - related genes in liver cancer samples by using TCGA liver hepatocellular carcinoma transcriptome data. With the exception of *UGT1A6*, all other genes were significantly less expressed in liver hepatocellular carcinoma as compared to normal liver samples (Fig. 5c). Furthermore, nicotine metabolism - related genes *CYP2A6*, *FMO3*, and *UGT2B7* were expressed at a similar level in benign cancer-matched normal livers as in normal liver controls. However, *CHRNA4* was significantly down-regulated in cancer-matched normal livers (Fig. 5c).

## Discussion

Nicotine is a lipophilic compound present at high levels in tobacco leaves, and can be easily absorbed in the bloodstream after smoking or chewing tobacco leaves. Nicotine can rapidly cross the blood-brain barrier and bind with high affinity to neuronal nicotinic acetylcholine receptors (nAChRs). nAChR activation excites target cells and mediates fast synaptic transmissions in autonomous ganglionic neurons in the brain [22, 23]. In human brain, *CHRNA4* and *CHRNB2,* in the form of (α4)_3_(β2)_2,_ are the most abundant subunits of pentameric neuronal nicotinic receptors; however, other nAChR subunits (α3, α5, α7, β2, β3, β4) also function as important components of homomeric/heteromeric receptor complexes [24]. All of these genes are associated with human smoking behaviors and nicotine addiction [6, 7, 25–33].

In an effort to define the tissue-specific epigenomic and transcriptomic landscape of nAChR genes, we discovered that *CHRNA4* was highly expressed in human liver. Additionally, *CHRNA3*, *CHRNA5*, and *CHRNB4* were highly expressed in colon and kidney. Expression of these neuronal-type nAChRs in non-neuronal tissues was strongly correlated with their tissue-specific epigenomic patterns. Further investigation led us to the discovery of a novel alternative promoter that regulates *CHRNA4* transcription specifically in liver. Importantly, this regulatory mechanism is evolutionarily conserved, as we confirmed an almost identical pattern in mouse. Our analysis further suggests that transcription factors *HNF4A* and *RXRA* may play a role as the upstream regulators of CHRNA4, potentially orchestrating the co-regulation of *CHRNA4* and *CYP2A6*, a key gene involved in nicotine metabolism [34]. Thus, our results establish correlated regulation as well as deregulation between *CHRNA4*, a gene that encodes a nicotine acetylcholine receptor, and genes involved in nicotine metabolism, in the context of normal liver and liver cancer, opening doors for questioning CHRNA4’s role in nicotine metabolism regulation. Considering the role of *CHRNA4* in mediating nicotine’s effect as a receptor, it is tempting to hypothesize that it might play a novel role as sensor in recognizing nicotine during its metabolism in liver (Fig. 5d). Although some evidence has suggested that individuals with reduced metabolic function of *CYP2A6* smoke fewer cigarettes and have a shorter smoking duration [35], the functionality of CHRNA4 in both normal liver and hepatocellular carcinoma still need to be further intensively investigated.

Smoking behavior is associated with liver cancer [21]. However, the molecular mechanism underlying liver cancer and the usage of tobacco, specifically nicotine metabolism, remains a mystery. We found that the expression level of *CHRNA4* and nicotine metabolism genes, including *CYP2A6*, *FMO3*, *UGT2B7* were dramatically down-regulated in human hepatocellular carcinoma, suggesting disrupted nicotine metabolism in hepatocellular carcinoma. Interestingly, *CHRNA4* expression was low in matched normal liver cells from patients with cancer. Our analysis put *HNF4A* upstream of liver-specific expression of both *CHRNA4* and *CYP2A6*, providing a potential mechanistic link between nicotine receptor and nicotine metabolism. *HNF4A* could be a key factor connecting nicotine metabolism and liver cancer. Previous studies showed that *HNF4A* was dramatically down-regulated or impaired in hepatocellular carcinoma [36, 37], and that forced expression of *HNF4A* in hepatocellular carcinoma cells could promote the transition of tumors towards a less invasive phenotype [38, 39]. Collectively, these findings suggest a potential connection between nicotine metabolism and liver cancer. Understanding the molecular mechanism of such a connection could facilitate the study of smoking-associated hepatocellular carcinogenesis, and might shed new lights on clinical therapy of smoking cessations and liver cancer.

## Conclusion

Nicotinic receptor genes are strongly associated with smoking behavior and nicotine dependence, and they are generally believed to be expressed specifically in the brain. In this work, by applying integrative genomics and comparative genomics, we described the expression and epigenetic landscape of nicotinic receptor genes in different non-neuronal human tissues. We found that nicotinic receptor alpha-4 (*CHRNA4*) was highly expressed in liver tissue, when comparing to brain and other tissues. We discovered a tissue-specific usage of an alternative *CHRNA4* promoter in human brain and liver, identifying a novel liver-specific transcription start site of *CHRNA4*, located about 3.9 KB upstream of known canonical RefSeq TSS. This tissue-specific, alternative promoter usage pattern is conserved in mouse, suggesting a dynamic but epigenetically conserved evolutionary history of *CHRNA4*. We also found that the expression level of *CHRNA4* was highly correlated with nicotine metabolism genes, and *CHRNA4* was down-regulated in both hepatocellular carcinoma and tumor-adjacent normal liver tissues. Our study indicated that the integrative analysis of published data could reveal new directions in investigating the molecular mechanisms in nicotine sensing and metabolism in liver, and how disruption of these processes may play a role in hepatocellular carcinogenesis.

## Methods

### Processing RNA-seq data of the Human Roadmap Epigenome Project

Processed mRNA-seq datasets (aligned to human reference genome hg19) from 56 reference epigenomics were obtained from Roadmap epigenomics project through data portal (http://egg2.wustl.edu/roadmap/web_portal/). Expression of all nAChRs were isolated and visualized by using the gplots package in the R environment (Ver 3.2.2).

### Processing RNA-seq data from the TCGA project

Processed mRNA-seq datasets (level 3, ht-seq reads count files) of 374 liver cancer samples and 50 cancer-matched normal samples were downloaded from the Genomic Data Commons Data Portal (https://gdc-portal.nci.nih.gov/). The RPKM of each gene was calculated based on the annotated human gene length (GENCODE V23).

### Processing RNA-seq data from the GTEx project

Processed mRNA-seq datasets (version V6, Reads Per Kilobase of transcript per Million mapped reads (RPKM) of genes) and data description files of 8555 samples were downloaded from the GTEx Portal (http://www.gtexportal.org/), including 119 liver samples, 320 lung samples, 149 colon sigmoid samples, 88 small intestine samples, and 1259 brain samples. Genes expression levels in different tissues (brain, liver, colon, small intestine, lung, and others) were plotted by using the ggplot2 package in the R environment.

### Co-expression correlation calculation

One-hundred nineteen human liver transcriptomes in GTEx V6 data were used to calculate the co-expression correlation between *CHRNA4* and other genes. Genes with an averaged expression level less than 1 RPKM were filtered out. The Pearson correlation coefficient between *CHRNA4* and all other genes was calculated by using log-transformed RPKM with the *cor* function, and *p*-values were calculated using the *cor.test* function in the R environment. *p*-values were further corrected using the *p.adjust* function with the BH method in R. Only the genes with an adjusted *p*-value less than 0.01 were considered as significantly correlated to *CHRNA4*, and were used to perform Ingenuity Pathway Analysis.

### Ingenuity pathway analysis (IPA)

IPA (Ingenuity Systems, Redwood City, CA) software was used to determine the functional pathways and regulatory network models represented by the significantly correlated genes. The gene set was imported into IPA to perform a Core Analysis. The top 15 enriched canonical pathways were selected based on significance (*p*-value < 0.05 ).

### ChIP-seq data preprocessing and peak calling

The raw reads of *RXRA* and *HNF4A* ChIP-seq data were downloaded from GEO, and aligned to the human genome (assembly hg19) and mouse genome (assembly mm9) using Bowtie V1.0.0 [40]. methylQA was used to process aligned bam files, isolate the non-redundant, uniquely aligned reads only, and extend the DNA fragments to 150 bp [41]. Additional file 8: Table S2 summarizes the information for the individual ChIP-seq data sample files used in this study.

The histone ChIP-seq data for human tissues (liver, brain, lung, colon and CD34-HSC) were obtained from the Roadmap Epigenomics Project through a data portal (http://egg2.wustl.edu/roadmap/web_portal/). Raw-reads were aligned to human genome (assembly hg19) and mouse genome (assembly mm9) by using Bowtie V1.0.0 [40]. methylQA was used to process aligned bam files, isolate the non-redundant, uniquely aligned reads only, and extend the DNA fragments to 150 bp [41].

The histone ChIP-seq data for mouse liver and cortex were obtained from the ENOCDE project through a data portal (https://www.encodeproject.org.). Raw-reads were aligned to the mouse genome (assembly mm9) using Bowtie V1.0.0 [40]. methylQA was used to process aligned bam files, isolate the non-redundant, uniquely aligned reads only, and extend the DNA fragments to 150 bp [41].

The MACSv2.0.10 [42] peak caller was used to compare ChIP-seq signal to a corresponding ChIP-seq input control. To identify narrow regions of transcription factor/histone enrichment (peaks) across the genome, a q-value threshold of 0.01 was used. The bedGraph transcription factor ChIP-seq data files and histone ChIP-seq data files were visualized on the WashU Epigenome Browser.

### FANTOM5 CAGE data processing

Cap Analysis Gene Expression by sequencing (CAGE-seq) data (bam files, liver and brain tissues for both human and mouse) generated by the FANTOM5 consortium were downloaded from FANTOM FTP (http://fantom.gsc.riken.jp/5/datafiles/latest). The bam files were then converted to a fastq file format, and aligned to human genome (assembly hg19) and mouse genome (assembly mm9) using Bowtie V1.0.0 [40]. The uniquely aligned reads were isolated using Samtools, and further transformed into bed files for visualization on the WashU Epigenome Browser.

### DNA methylation data processing

Methylation calls for each CpG site were calculated using Whole-Genome Bisulfite Sequencing (WGBS) data for human tissues (liver, brain, lung, colon and CD34-HSC) obtained from the Roadmap Epigenomics Project through a data portal (http://egg2.wustl.edu/roadmap/web_portal/), and were visualized on the WashU Epigenome Browser. To measure the DNA methylation level of *CHRNA4* promoters, methylation of CpG sites with a minimum of 10× coverage per site in a 1 KB region around the *CHRNA4* TSS in human liver, brain, and CD34-HSC were isolated to generate boxplots and calculate statistical significance.

### eQTL data processing

Tissue-specific eQTL data were downloaded from the GTEx Portal (http://www.gtexportal.org/). The SNPs located in CHRNA4 loci and associated with CHRNA4 (ENSG00000101204.11) in human liver and hippocampus were isolated using an in-house python script. The *p*-value of each SNP was negatively log-transformed and visualized on the WashU Epigenome Browser.

### Motif analysis

Motif analyses were performed using the FIMO tool from the MEME suite [43]. The 10 bp upstream and downstream each SNP were isolated using bedtools (getfasta) from the human reference genome (assembly hg19). Two allele-specific 21 bp DNA sequences were generated based on the allelic information obtained from dbSNP (build 144). Fimo was used to predict potential TF binding sites in two allele-specific 21 bp DNA sequences by using a PWM of 519 transcription factors downloaded from the JASPAR database [44].

### Genome alignment

A genome alignment generated by blastz between human (hg19) and mouse (mm9) was obtained from the UCSC genome browser (http://hgdownload.cse.ucsc.edu/downloads.html), and then visualized on the WashU Epigenome Browser to indicate the genome-level conservation at the CHRNA4/Chran4 loci. Multiple alignments of 45 vertebrate genomes of CHRNA4 promoters were directly generated by UCSC genome browser (http://hgdownload.cse.ucsc.edu/).

### Quantitative real time PCR (qRT-PCR) analysis

The qRT-PCR analyses were performed using the SuperScript VILO cDNA Synthesis Kit (Life Technologies) with iTaq Universal SYBR Green Supermix (Bio-Rad). All mouse and human brain and liver RNA was purchased from ZYAGEN. 500 ng total RNA was used in a 20ul reverse transcription reaction. The cDNA obtained was diluted to a total volume of 100ul and stored at −20 °C. The primers for human *CHRNA4* and mouse *Chrna4* (listed in Additional file 8: Table S3) were synthesized by Integrated DNA Technologies. The qRT-PCR was performed in a 20ul reaction mixture consisting of 2ul diluted cDNA, 0.2uM of each primer, and 10ul iTaq Universal SYBR Green Supermix. All amplifications were carried out in a Bio-Rad CFX96 Real-Time PCR Detection (Bio-Rad) with denaturation at 95 °C for 30s, followed by 40 cycles at 95 °C for 5 s and 60 °C for 30s. A melting curve analysis was performed for each run to confirm the specificity of amplification and lack of primer dimers. The qRT-PCR experiments were always run in triplicate. The relative mRNA expression levels of target genes were quantified using the 2-ΔΔCT methods as reported [45].

## Additional files

Additional file 1: Figure S1. The epigenetic landscape around CHRNB4, CHRNA5, CHRNA3, CHRNB2, and CHRNA4 in human liver, CD34-HSC, brain, colon, and lung tissues. (PDF 288 kb)

Additional file 2: Figure S2. The epigenetic landscape and expression pattern of Chrna4 in mouse brain and liver. (PDF 115 kb)

Additional file 3: Figure S3. Transcription factors binding events around CHRNA4 promoter. (PDF 33.3 kb)

Additional file 4: Figure S4. Distribution of expression correlation coefficient between CYP2A6 and all genes in 119 human liver samples. (PDF 44.9 kb)

Additional file 5: Table S1. List of genes significantly correlated with CHRNA4 in human liver. (XLSX 123 kb)

Additional file 6: Figure S5. Enriched network modules in genes positively correlated to CHRNA4. (PDF 290 kb)

Additional file 7: Figure S6. The absolute expression level of CHRNA4, CYP2A6, UGT1A6, UGT2B7, and FMO3 in 119 human liver samples. (PDF 49.9 kb)

Additional file 8: Table S2-3. Dataset and primers used in this study. (DOCX 68.5 kb)

## Abbreviations

CAGE-seq: Cap Analysis Gene Expression sequencing
CD34-HSCs: CD34+ Hematopoietic Stem Cells
eQTL: Expression Quantitative Trait Loci
GTEx: Genotype-Tissue Expression project
nAChRs: Nicotinic acetylcholine receptors
RPKM: Reads Per Kilobase of transcript per Million mapped reads
SNPs: Single Nucleotide Polymorphisms
TSS: Transcription Start Site
