# Supplementary material for: Uncovering the transcriptomic and epigenomic landscape of nicotinic receptor genes in non-neuronal tissues
Source: BMC Genomics. 2017 Jun 5;18:439. doi: 10.1186/s12864-017-3813-4 (PMC5460472; doi:10.1186/s12864-017-3813-4)

Supplementary figure1. Epigenetic landscape around CHRNB4, CHRNA5, CHRNA3, CHRNB2, and CHRNA4 in human liver, CD34-HSC, brain, colon, and lung tissues.

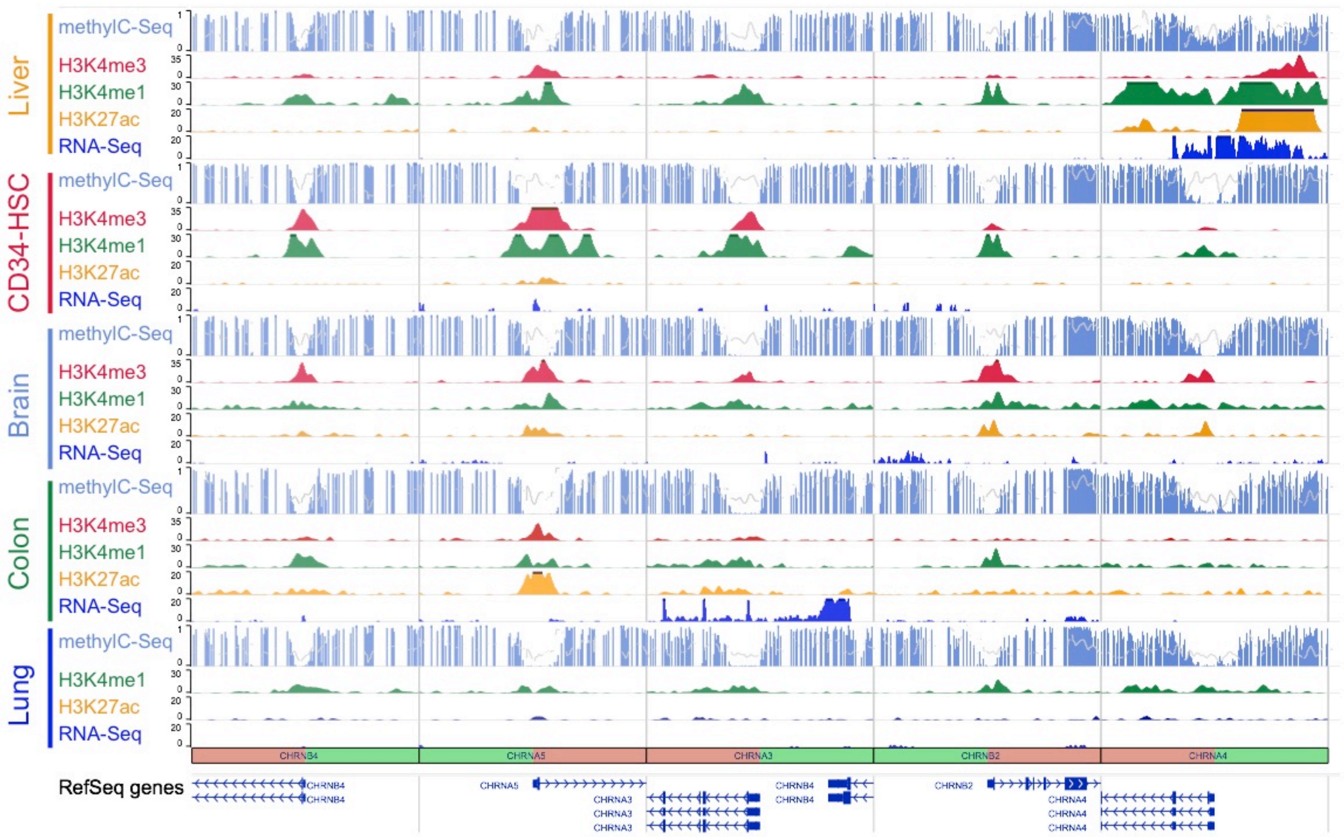

Supplement: Supplementary file 1 — The epigenetic landscape around CHRNB4, CHRNA5, CHRNA3, CHRNB2, and CHRNA4 in human liver, CD34-HSC, brain, colon, and lung tissues. (PDF 288 kb) [file 12864_2017_3813_MOESM1_ESM.pdf]
