# Supplementary material for: Uncovering the transcriptomic and epigenomic landscape of nicotinic receptor genes in non-neuronal tissues
Source: BMC Genomics. 2017 Jun 5;18:439. doi: 10.1186/s12864-017-3813-4 (PMC5460472; doi:10.1186/s12864-017-3813-4)

Supplementary figure 2. A). The RefSeq promoter of Chrna4 (P1, pink-shaded) is enriched for the H3K4me3 histone modification and RNA polymerase II ChIP-seq signal (Pol2) in mouse cortex. The CAGE-seq signal of cortex indicates Chrna4 is transcribed from the RefSeq promoter P1. The liver-specific promoter (blue-shade), which is located ~4.8KB upstream of the RefSeq promoter, is enriched for strong, active histone modifications and RNA polymerase II ChIP-seq signal (Pol2). The CAGE-seq signal of liver indicates Chrna4 is only transcribed from the liver-specific promoter P2. **B).** qRT-PCR measured relative expression of Chrna4 in mouse brain and liver tissues.

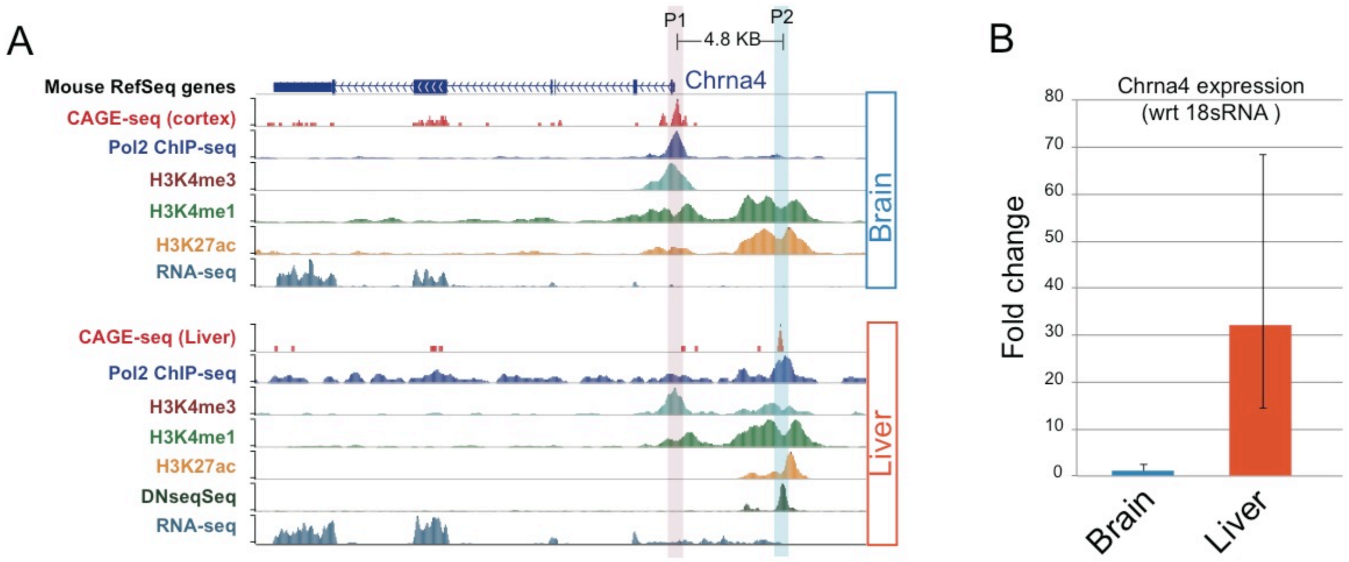

Supplement: Supplementary file 2 — The epigenetic landscape and expression pattern of Chrna4 in mouse brain and liver. (PDF 115 kb) [file 12864_2017_3813_MOESM2_ESM.pdf]
