# Supplementary figures and images for: Uncovering the transcriptomic and epigenomic landscape of nicotinic receptor genes in non-neuronal tissues
Source: BMC Genomics. 2017 Jun 5;18:439. doi: 10.1186/s12864-017-3813-4 (PMC5460472; doi:10.1186/s12864-017-3813-4)

Supplementary figure 3. Transcription factors binding events around CHRNA4 promoter.

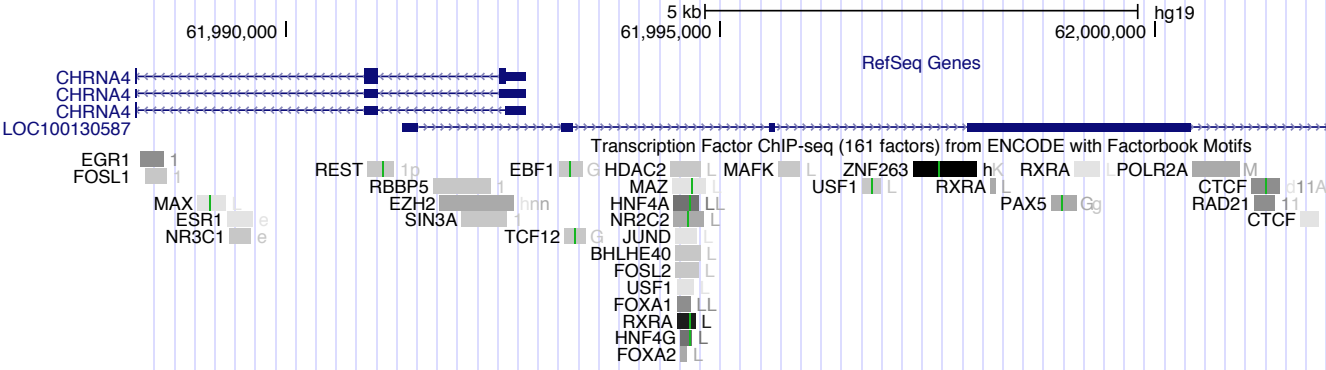

Supplement: Supplementary file 3 — Transcription factors binding events around CHRNA4 promoter. (PDF 33.3 kb) [file 12864_2017_3813_MOESM3_ESM.pdf]
