# Supplementary material for: Uncovering the transcriptomic and epigenomic landscape of nicotinic receptor genes in non-neuronal tissues
Source: BMC Genomics. 2017 Jun 5;18:439. doi: 10.1186/s12864-017-3813-4 (PMC5460472; doi:10.1186/s12864-017-3813-4)

Supplementary figure4. Distribution of expression correlation coefficient between CYP2A6 and all genes in 119 human liver samples. Red line: expression correlation coefficient between CYP2A6 and CHRNA4.

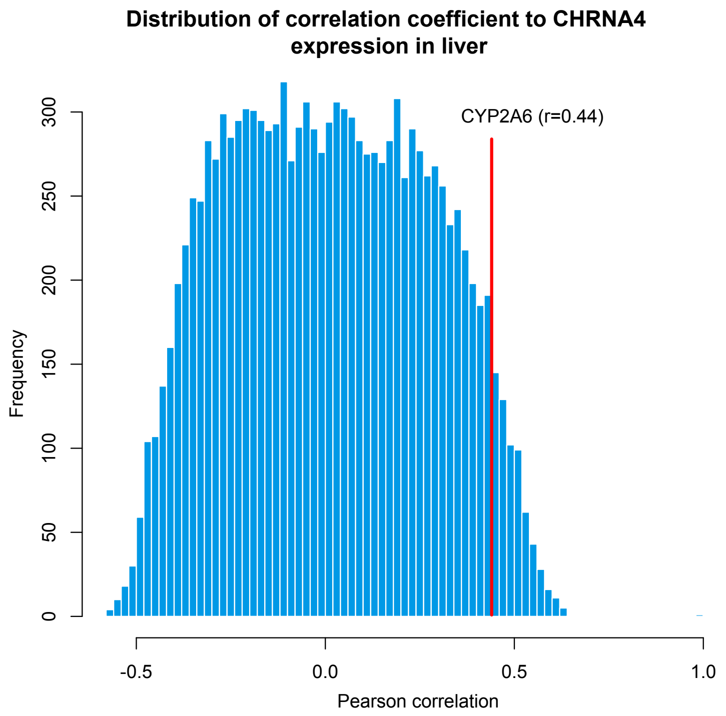

Supplement: Supplementary file 4 — Distribution of expression correlation coefficient between CYP2A6 and all genes in 119 human liver samples. (PDF 44.9 kb) [file 12864_2017_3813_MOESM4_ESM.pdf]
