# Supplementary material for: Uncovering the transcriptomic and epigenomic landscape of nicotinic receptor genes in non-neuronal tissues
Source: BMC Genomics. 2017 Jun 5;18:439. doi: 10.1186/s12864-017-3813-4 (PMC5460472; doi:10.1186/s12864-017-3813-4)

Supplementary figure5. IPA identified top3 enriched network modules in genes positively correlated to CHRNA4 in 119 human liver samples.

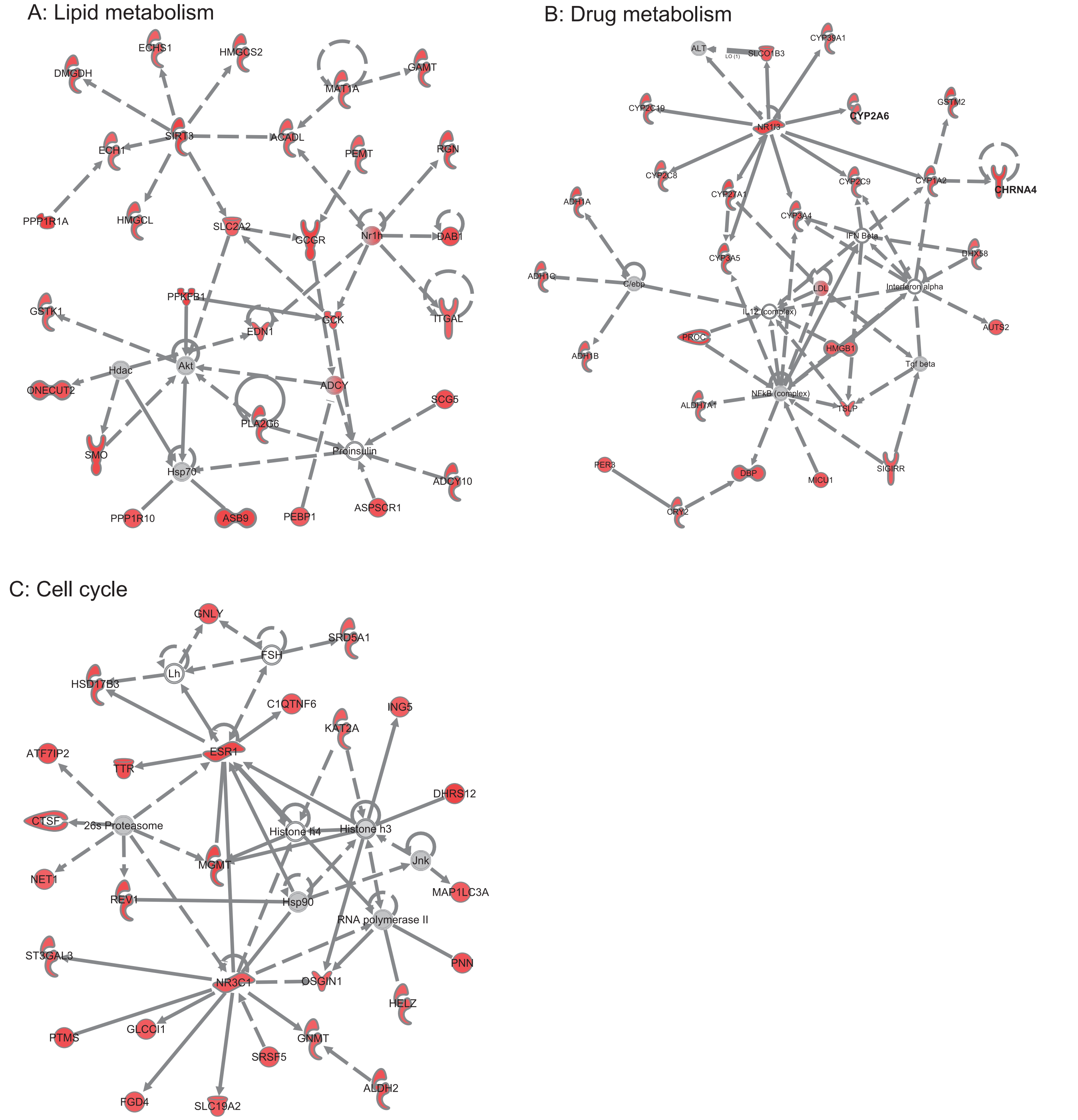

Supplement: Supplementary file 6 — Enriched network modules in genes positively correlated to CHRNA4. (PDF 290 kb) [file 12864_2017_3813_MOESM6_ESM.pdf]
