# Supplementary material for: Uncovering the transcriptomic and epigenomic landscape of nicotinic receptor genes in non-neuronal tissues
Source: BMC Genomics. 2017 Jun 5;18:439. doi: 10.1186/s12864-017-3813-4 (PMC5460472; doi:10.1186/s12864-017-3813-4)

Supplementary figure 6. Absolute expression level of CHRNA4, CYP2A6, UGT1A6, UGT2B7, and FMO3 in 119 human liver samples

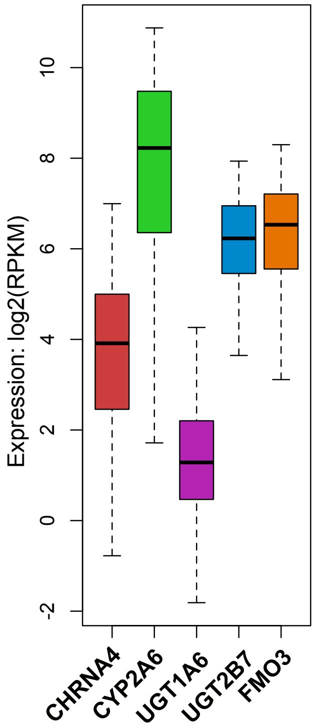

Supplement: Supplementary file 7 — The absolute expression level of CHRNA4, CYP2A6, UGT1A6, UGT2B7, and FMO3 in 119 human liver samples. (PDF 49.9 kb) [file 12864_2017_3813_MOESM7_ESM.pdf]
