# Supplementary material for: Uncovering the transcriptomic and epigenomic landscape of nicotinic receptor genes in non-neuronal tissues
Source: BMC Genomics. 2017 Jun 5;18:439. doi: 10.1186/s12864-017-3813-4 (PMC5460472; doi:10.1186/s12864-017-3813-4)
Supplement: Supplementary file 8 — Dataset and primers used in this study. (DOCX 68.5 kb) [file 12864_2017_3813_MOESM8_ESM.docx]

**Supplementary -Table 2. TF ChIP-seq dataset used in study**

| Transcription factor | Dataset GEO accession |
| --- | --- |
| HNF4A | GSE31477 |
| RXRA | GSE31477 |
| Hnf4a | GSE22078 |
| Rxra | GSE53736 |

**Supplementary -Table 3-A. qRT-PCR primers of human CHRNA4**

| Primer | Primer sequence |
| --- | --- |
| human_CHRNA4_F | CGGACATCGTCCTCTACAACA |
| human_CHRNA4_R | GGTCGAAGGGGAAGAAGGTG |
| 18S_rRNA_F | GTAACCCGTTGAACCCCATT |
| 18S_rRNA_R | CCATCCAATCGGTAGTAGCG |

**Supplementary -Table 3-B. qRT-PCR primers of mouse Chrna4**

| Primer | Primer sequence |
| --- | --- |
| mouse_Chrna4_F | TCCGCATCCCATCTGAACTC |
| mouse_Chrna4_R | AGAACAGGTGGGCTTTGGTT |
| 18S_rRNA_F | GTAACCCGTTGAACCCCATT |
| 18S_rRNA_R | CCATCCAATCGGTAGTAGCG |
